# Gestational Diabetes Mellitus in a Multi-Ethnic, High-Risk Population: Adequacy of Screening for Diabetes Mellitus 6 Weeks after Delivery

**DOI:** 10.3390/ijerph192113946

**Published:** 2022-10-27

**Authors:** Mukesh M. Agarwal, Madan Lal, Chintan D. Vyas

**Affiliations:** 1 Department of Pathology, Faculty of Medicine, California University of Science and Medicine, Colton, CA 92324, USA; 2 Department of Obstetrics and Gynecology, Saint Luke’s General Hospital, R95 FY71 Kilkenny, Ireland; 3 Department of Medicine, Burjeel Medical Center, Al Ain P.O. Box 103500, United Arab Emirates

**Keywords:** gestational diabetes, follow-up, Arabs, OGTT

## Abstract

Gestational diabetes mellitus (GDM) during pregnancy is a marker for future type 2 diabetes mellitus (T2DM); therefore, a meticulous follow-up after delivery can help identify women at risk for T2DM. In a cohort of 5504 pregnant women, the postpartum follow-up of all 1043 women with GDM for hyperglycemia in a multi-ethnic, high-risk Arab population was investigated. The prevalence of GDM was 18.9%. A total of 265 (25.4%) women returned for an oral glucose tolerance test (OGTT) 4–6 weeks after delivery, with more South Asian than Arab women (*p* < 0.01). The other factors associated with return were (a) family history of T2DM, (b) lower basic metabolic index, (c) higher abortions and (d) lower gravida (*p* < 0.05), all with minimal effect. An abnormal postpartum OGTT was statistically associated with previous GDM history and hypoglycemic drug treatment, although these effects were small. Overall, the follow-up of women with GDM postpartum was dismal, ethnicity being the major factor influencing return. Urgent public measures are needed to educate women with GDM about follow-up highlighting (a) risk awareness for T2DM and (b) a healthy lifestyle after childbirth—if we are to turn the tide on the epidemic of T2DM plaguing the Arab world.

## 1. Introduction

Over 50 years ago, O’Sullivan and Mahan’s original diagnostic criteria for gestational diabetes mellitus (GDM) were formulated to predict type 2 diabetes mellitus (T2DM) after delivery [1]. However, over the years, the focus of GDM has changed; it is mainly used in predicting and preventing the many associated maternal (e.g., preeclampsia, caesarean sections, birth injuries) and fetal (e.g., macrosomia, hypoglycemia, shoulder dystocia) complications in index pregnancy via treatment [2,3]. This change often misses the much wider and farsighted picture [4]. After childbirth, women with GDM have up to 7–10-fold increase in developing T2DM as substantiated by multiple systemic reviews and meta-analyses [4,5,6]. GDM also predisposes to other problems after delivery such as metabolic syndrome [7] and cardiovascular disease [8]. Therefore, all preeminent medical associations such as the American Diabetes Association [9] recommend screening women with a history of GDM for type 2 DM approximately 4–12 weeks after delivery and lifelong every 3 years thereafter.

T2DM and GDM are exponentially increasing worldwide with T2DM reaching epidemic proportions. It is well-established that the impending T2DM after childbirth can be forestalled by strict preventive measures (diet, exercise, drugs) after childbirth [10]. Thus, several opportunities are available for limiting T2DM in women with a history of GDM, by education about risk awareness, implementation of a healthy lifestyle, breast-feeding and pharmacotherapy as shown by the Diabetes Prevention Program, which reduced the incidence of T2DM by 40% [11].

The United Arab Emirates (UAE) is a multi-ethnic, multi-racial country where the prevalence of T2DM (approximately 20.1%) is amongst the highest in the world [12]. For years, the T2DM prevalence in UAE was the second highest in the world. The prevalence of GDM in the UAE varies from 7.9 to 37.3%, depending on the criteria used for the diagnosis [13]. In general, across the world, follow-up of women with GDM after delivery is not good, varying widely from approximately one to four women for every five women [14]. Postpartum care remains a major challenge, and low levels of awareness are present across the world [15]. If follow-up after GDM is not excellent, a great opportunity to change the epidemiology of T2DM in the UAE would be missed [16].

In general, follow-up studies after GDM from the Middle East are almost non-existent as pointed out by a metanalysis of 39 cohort studies [6]. The aim of this study was to document how good is the follow-up after GDM in a major obstetric hospital in the UAE. The findings could serve as a microcosm of the problems affecting the country at large, and it is potentially possible to extrapolate the findings to similar affluent Arabian Gulf countries. Furthermore, it would be possible to address issues such as: How to good is follow-up? How can the follow-up be improved if poor? How to motivate women with GDM to follow lifestyle changes after delivery so that we can prevent future impending T2DM.

## 2. Materials and Methods

### 2.1. Subjects

The subjects were pregnant women with singleton pregnancies routinely attending antenatal clinics of a 200-bed obstetric hospital in Al Ain, United Arab Emirates. Currently, approximately 3000 women are delivered per year. As part of a universal screening program, all women are screened for GDM.

All women with the discharge diagnosis of GDM were identified from the hospital electronic medical record database between January 2013 and December 2014. The incidence of T2DM is maximum after 5–6 years [16], so an older time frame is ideal to obtain a better picture. However, due to almost no follow-up after 6 weeks, longer follow-up could not be studied.

The following epidemiological data were collected from each medical record: age, nationality (9 categories), gestational age at OGTT, history of previous GDM, family history of GDM, gravidity, parity, body mass index (BMI), birth weight, delivery method (3 categories: normal vaginal, assisted vaginal and cesarian section delivery), gestational age at delivery, treatment (4 categories).

### 2.2. Antenatal GDM Diagnosis

The hospital follows a 1-step approach for the diagnosis of GDM, i.e., all women are screened by the 75 g OGTT at 24–28 weeks gestation. Since 2011, the IADPSG criteria are used for diagnosis (i.e., GDM is diagnosed if any one value of the 75 g OGTT is ≥ 92, 180, 153 mg/dL at 0 h, 1 h and 2 h, respectively), which was the ‘gold standard.’

### 2.3. Postpartum OGTT Diagnostic Categories

For the 75 g OGTT, the American Diabetes Association criteria [9] were used to define impaired glucose tolerance, IGT (2 h glucose = 140–199 mg/dL), impaired fasting glucose, IFG (fasting glucose = 100–125 mg/dL, and DM (fasting glucose ≥ 126 mg/dL and/or 2 h value ≥ 200 mg/dL).

### 2.4. Data Analysis

Data were logged into a computer database and analyzed by SPSS ver. 26 for Windows (IBM Inc., Armonk, NY, USA).

The distribution of continuous variables was tested for normality by the Shapiro–Wilk test and presented as mean or median. Categorical data are presented as a percentage. Nonparametric tests were used since the data were not normally distributed. The level of statistical significance was set at *p* < 0.05. Associations between postpartum impaired glucose tolerance, IGT/T2DM and family history, insulin, and drugs were tested using a χ^2^ test, while age and BMI were tested using the Mann–Whitney test. Cramer’s V correlation was also used in tables with more than 2 × 2 rows and columns. The relationship between postpartum IGT/T2DM and patient characteristics (age, parity, BMI, family history of T2DM, gestational age at delivery, drugs/insulin use) was assessed using both univariate and multivariate logistic regression.

Analysis of the variance was used to find the differences in group means between women (who returned vs. who did not) for an OGTT and those with normal OGTT to those with impaired (IGT/IFG) or seriously impaired OGTT (T2DM). If a variable violated the assumption of homogeneity of variance, Welch’s F (FW) was used.

## 3. Results

### 3.1. Patients

There were 5684 deliveries in the hospital during the study time-period (3215 and 2469 deliveries in 2013 and 2014, respectively; of these, 1223 women who were identified with the diagnosis of GDM during their antenatal visits. However, 180 women had to be excluded as follows: (1) 5 (0.41%) women with twins, (2) 18 (1.47%) women, who had pre-existing type 2 DM, and (3) 157 (12.8%) women who delivered in other hospitals due to either changing the health providers after initial follow-up at our hospital or moving out of the city or country. After excluding these 180 women from the analysis, the study cohort consisted of 5504 deliveries, and 1043 women with GDM were used in the final analysis.

### 3.2. Prevalence of Gestational Diabetes

During the two-year study period, there were 5504 deliveries of which 1043 women had GDM. Thus, the prevalence of GDM using IADPSG criteria was 18.9%, which is similar to other studies from the UAE [12].

### 3.3. Maternal Age and Gestational Age

The mean maternal age was (mean ± SD) 30.68 ± 5.8 years. The mean gestational age (at time of OGTT) was 24.2 ± 7.78 weeks.

### 3.4. Nationality of the Cohort

The major ethnic groups of the study population were as follows: 869 (94.7%) women were Arabs; 106 (10.2%) women were from the Indian subcontinent (India, Pakistan, Bangladesh and Sri Lanka); 58 (5.6%) women identified with ‘other’ nationalities from multiple diverse countries. These 58 women included 32 (3.1%) women from the Philippines, 16 (1.5%) women from the United Kingdom, Canada, Holland and 10 (1.0%) women from Iran. The nationality of 10 (1.0%) women was unknown. Of the Arabs, 729 (69.9%) women were Asian Arabs (UAE, Saudi Arabia, Oman, Kuwait, and Bahrain), 79 (7.6%) women were Shami Arabs from Syria, Palestine, Jordan, and Lebanon, 25 (2.4%) women were East African Arabs from Somalia and Sudan, and 36 (3.5%) women were North African Arabs from Egypt, Tunisia, and Algeria.

### 3.5. Type of Delivery

In the cohort of 1043 women, 808 (77.5%) women underwent a normal delivery, 5.0 (0.5%) women underwent an assisted vaginal delivery, and 230 (22.1%) women underwent a cesarian section.

### 3.6. Follow-Up of Gestational Diabetes

Of the entire cohort of 1043 women diagnosed with GDM, 778 (74.6%) women did not return for an OGTT; 265 (25.4%) women returned and underwent an OGTT at 4–6 weeks postpartum. Of these 265 women, 210 (79.2%) women had an OGTT within normal limits, while 55 (20.8%) women) had an abnormal OGTT: 10 (3.8%) women had DM2, 25 (9.4%) women had IGT, and 20 (7.5%) women had IFG.

### 3.7. Characteristics of Women Who Retuned for an OGTT

The patients who returned showed a significant association with the following (Table 1): (a) nationality, χ^2^ (8) = 49.84, *p* < 0.01. Using the standardized residual values, significantly more women from South Asia (*p* < 0.01) and the Philippines (*p* < 0.01) returned for an OGTT, while fewer women from the rich Arab states (UAE/Oman/Saudi Arabia) (*p* < 0.01). (b) women with family history of DM, χ^2^ (1) = 5.06, *p* = 0.02, and (c) lower BMI, χ^2^ (3) = 8.70, *p* = 0.03 did not return. Although both family history of DM and BMI were statistically significant, the standardized residuals did not clearly show where the significance lay (all < 1.96), and the value for Cramer’s V was 0.07 and 0.09, respectively, indicating a very small effect for both these variables; (d) gravida: The average number of pregnancies (i.e., gravida) for the group who did not return was significantly greater (*p* < 0.01) than that observed in the group who returned (e) Abortions: The average number of abortions the group who did return was significantly greater (*p* = 0.04) than that observed in the group who did not return. However, both number of pregnancies (gravida) and number of abortions violated the assumption of homogeneity of variance. The patients who returned showed no significant association with the following: (a) chronological age χ^2^ (3) = 1.67, *p* = 0.79. (b) previous GDM χ^2^ (3) = 3.92, *p* = 0.24; (c) delivery method χ^2^ (2) 3.64, *p* = 0.13.

### 3.8. Characteristics of Women with Abnormal OGTT

There was a significant association between abnormal OGTT results with the following (Table 2): (a) previous GDM, χ^2^ (6) = 28.14, *p* < 0.01. Using standardized residuals, significantly more women with previously diagnosed GDM had T2DM more than expected. The Cramer’s V of 0.23 indicated a small effect (Fisher’s exact test = 12.06, *p* = 0.07); (b) drug/treatment intervention, χ^2^ (4) = 11.16, *p* = 0.02. Using standardized residuals, significantly more patients with DM were being treated with insulin than expected (*p* < 0.01). The value for Cramer’s V of 0.14 indicated a small effect (Fisher’s exact test = 9.28, *p* = 0.04).

As expected, three variables were significantly different: fasting glucose (FW (2, 14.16) = 24.83, *p* < 0.01) followed by glucose at 1 h (F (2, 29) = 10.90, *p* < 0.01) and glucose at 2 h (F (2, 138) = 93.22, *p* < 0.01). The glucose levels for 1 h were significantly higher in the DM group (mean = 218.00, SD = 11.31) when compared to the normal group (mean = 131.20, SD = 24.50) (*p* = 0.02). There were no significant differences between DM and the IGT/IFG group (mean = 172.79, SD = 48.01) (*p* = 0.12) or the IGT/IFG and the normal group (*p* = 0.14) for the 1 h glucose.

Due to the difference in group sizes, post hoc analyses were performed using Games–Howell method. For fasting plasma glucose, the IGT/IFG (mean = 100.40, SD = 8.39) was the only group to significantly differ from the normal group (mean = 90.3, SD = 5.5) (*p* < 0.01). The DM group (mean = 127.2, SD = 36.8) was not significantly different than the normal (*p* = 0.08) or the IGT/IFG group (*p* = 0.21). Finally, at 2 h, significant differences between all groups were observed in the plasma glucose of the OGTT. The DM group (mean = 215.9, SD = 24.05) had significantly higher glucose levels than the normal group (mean = 105.6, SD = 18.43) (*p* > 0.01) and the IGT/IFG group (mean = 131.7, SD = 20.94) (*p* < 0.01). Similarly, the IGT/IFG group had significantly higher glucose levels than the normal group (*p* < 0.01).

## 4. Discussion

The major finding of this study is that the follow-up of GDM women postpartum at 6 weeks was dismal; only one-quarter of women with GDM returned for an after delivery OGTT. These women tended to have a lower BMI, were less likely to have a family history of DM, and were more likely to have a smaller number of pregnancies (gravid), and more abortions, although the effect was small. They were also likely to be from South Asian or Filipino ethnicity; the women from the richer Arab countries (UAE, Saudi Arabia, Oman) were less likely to return for an OGTT.

According to the hospital guidelines, all women with the diagnosis of GDM are enrolled for a 75 g OGTT, 6 weeks postpartum. Since most patients are Arabs, an Arabic-speaking nurse informs the patient about the result. If the OGTT is abnormal, an appointment with the diabetologist is made for treatment and follow-up of hyperglycemia. All GDM women are advised lifestyle preventive measures (a) in the later part of pregnancy during regular obstetric visits and (b) postpartum during the visits to the pediatrician ‘well-baby’ clinics. These women are asked to repeat the OGTT periodically (yearly) to find if they have converted to T2DM at each postpartum visit. However, only 23 women returned for after one or more years; all of them had conceived again. Clearly hospital guidelines are not followed—a problem that needs to be solved.

### 4.1. The Problem: Poor Gestational Diabetes Mellitus Postnatal Follow-Up

However, in general, follow-up testing after delivery of women with GDM remains poor universally with a wide variation [17,18]. Postpartum screening has increased over the last decade, but it is still suboptimal as confirmed by a cohort study of 14,448 GDM pregnancies delivered between 1995 and 2006 in California [19]. In some Asian studies, it has been reported to be as low as 13.1% [20]. In the UK, of the 788 females, 146 (18.5%) had glucose testing within the 6-month follow-up period [17]. In Finland, the overall return rate for postpartum testing in women was 35.7%, which included women in primary and tertiary care centers [21]. In a US hospital setting with an almost exclusive Hispanic population, only 19.8% of the 400 participants who completed screening completed it within 4–6 weeks of delivery [22]. In a Brazilian study, (51.7%) returned to be tested by 12 weeks [23]. In a study from Belgium, of 191 women with GDM, 70.7% attended the postpartum OGTT. They had a higher BMI, were more often from an ethnic minority, and smoked more often during pregnancy [24]. In a study on US national laboratory (Quest diagnostics) found that only 19% of women 23,299 women with GDM had postpartum glucose screening of any type within six months of delivery [25]. However, due to concentrated efforts, postpartum screening is improving in some countries.

Longer follow-ups are also poor. A UK study showed a deterioration of yearly follow-up over a 17-year period. The proportion of women tested in any given year averaged 34.2%; there was a progressive decline in the proportion of women receiving a yearly test with time since delivery [26].

### 4.2. Ethnicity and Postpartum Follow Up

Ethnicity was the most important factor in determining the follow-up of women with GDM, as seen in this study. In a study from the UK, a follow-up of 10,868 women with GDM, South Asian women had a significantly greater likelihood of being screened compared to White women within the first 5 years postpartum [5]. A comparison of attitudes to GDM in Middle Eastern women compared to Swedish women is revealing. Women from the Middle East thought GDM would disappear after delivery without follow-up while Swedish women feared developing T2DM, more often sought help, and used medications against pregnancy-related complications [27].

In another large study on nearly half a million insured women, at 12 weeks postpartum, 18% Asian compared with 12% White women were screened [15]. Compared to whites, Hispanic and Asian women have higher rates of GDM prevalence. Thus, ethnicity plays an important role in the follow-up response of women.

### 4.3. Characteristics of Women Who Returned

Women with GDM who returned for follow-up after delivery varied across studies with major inconsistencies [22]. In this study, women with lower BMI, no family history DM, a higher number of pregnancies (i.e., gravida), and increased abortions in the past tended to return, even though the effect was small. Some studies show that age has no bearing like this study [22].

### 4.4. Factors Responsible for Conversion to Type 2 DM

Several studies have identified factors associated with future risk of T2DM. Maternal age, pre-pregnancy weight and BMI, ethnicity, and a family history of DM are associated with conversion to type 2 DM after GDM. A study with a follow-up for 10 years after delivery from Sri Lanka showed [28] that maternal age at delivery ≥ 30 years, birth weight of the index child > 3.5 kg and treatment with insulin during the index pregnancy were the best predictors of T2DM. There was no significant association between future risk of T2DM and history of GDM in a previous pregnancy, family history of T2DM, parity, or gestational age at delivery. The risk factors for progression to T2DM include maternal age, prepartum and postpartum BMI, family history of T2DM, receipt of insulin for gestational diabetes, and fasting glucose level during pregnancy [29]. An in-depth knowledge of the epidemiology of GDM in different countries and regions will help to understand the disparities and similarities facilitating international comparisons. All this will help in our march toward our ultimate goal: preventing maternal and fetal complications in every index pregnancy and DM in the entire population. Rates of conversion to T2DM vary by population and range from 2 to 12.5% within one-year postpartum [30]. In this study, at 6 weeks, 55 (20.8%) women had impaired glucose tolerance, while 10 (3.8%) had T2DM.

### 4.5. Overcoming Barriers to Follow-Up GDM

The barriers to follow-up in Arab countries can only be extrapolated from countries with available data [14]. These impediments have been cited as follows: (a) patient lost to follow-up; (b) health care provider (in the obstetric setting) not seeing the patient; (c) patient not considering that follow-up tests are necessary after delivery; (d) stress of adjusting to a new baby; and (e) feeling healthy postpartum. All these aspects will help in planning effective strategies. The similarity in problems affecting many Arab countries calls for some major decisions. Updating screening guidelines to the latest research will help to reduce these differences. More education for the patients and the caregivers, especially in rural settings, would be a major step in the right direction. A survey of women in Canada [31] on their perceived status within 3–5 years of a pregnancy (with and without GDM) is telling. When questioned about their risk assessment, 47% of the women with a history of GDM believed that it was “very possible” that they would develop T2DM, whereas 35% believed it was “somewhat possible.” Thus, women with GDM can be motivated for follow-up, which is a reason to be optimistic.

GDM follow-up is characterized by communication where general practitioners appear to be information seekers whose communication needs are not met by hospital clinicians. Midwives are ideally placed to assist in improving communication and postnatal GDM follow-up [32].

In the Arab world, the ubiquitous fatalism is an additional cultural factor. Although GDM is a well-established risk factor for T2DM, many women with GDM are unaware of this increased risk, which might affect compliance with risk-reduction recommendations. A comparison of attitudes to GDM in Middle Eastern women compared to Swedish women is revealing. Women from the Middle East thought it would disappear after birth, while Swedish women feared developing Type 2 T2DM, more often sought help, and used medications against pregnancy-related complications [33].

Electronically generated telephone and e-mail reminder messages to patients may improve the rates of postpartum testing for persistence of glucose intolerance [30]. Thus, these are some of the important ideas that we plan to institute in our hospital.

## 5. Strengths and Limitations

A major strength of this study is that it is one of the few studies available from the Arab world, where T2DM is rampant. It can become the harbinger for more studies and should help the health authorities in the UAE to actively work on strategies of follow-up. The weaknesses are that it was a relatively small study and so no definite, clear-cut conclusions could be drawn to associate factors involved in return for T2DM testing and conversion to T2DM, and the association was small. Furthermore, we investigated only a few risk factors (such as BMI, previous GDM) for the diagnosis of T2DM in the postpartum period; however, many others have been identified in the literature such as postpartum triglycerides, history of no breast feeding, previous polycystic ovarian syndrome and higher education [34]. This was due to the retrospective nature of our study.

## 6. Conclusions

The follow-up after GDM in this study remains very poor. Ethnicity is an important factor showing an association with chances that a patient would return for follow-up. It is imperative to follow women with GDM after delivery, with all possible strategies, in the UAE and in the Arab world. Every woman with GDM must be exhorted to implement lifestyle changes strictly; if we can succeed, the avalanche of the expected future T2DM can be mitigated.

## Figures and Tables

**Table 1:** Comparison of women who did and did not return for an oral glucose tolerance test.

	Returned (Mean ± SD)	Did Not Return (95% CI)	*p* Value	Comments
*n* (%)	265 (25.4%)	778 (74.6%)		
Age (years)	30.8 (30.1–31.5)	30.63 (30.22–31.04)	NS	
FH DM	49.1%	57.0%	0.02	Cramer’s V 0.07; very small effect
BMI	28.4 (28.0–29.4)	29.20 (28.78–29.61)	0.03	Cramer’s V 0.09; very small effect
Previous GDM	68.3%	38.2%	NS	
Gravida	3.3 (3.04–3.58)	3.9 (3.7–4.1)	0.01	homogeneity of variance violated
Abortions	2.02 (1.54–2.50)	1.68 (1.50–1.85)	0.04	homogeneity of variance violated
Birth weight	3242.1 (3041.3–3442.8)	3164.5 (3076.07–3252.90)	NS	
OGTT week	23.9 (22.9–24.8)	24.23 (23.7–24.8)	NS	
Gestational age at delivery (weeks)	38.3 (34.0–42.8)	38.42 (34.7–41.9)	NS	

FH DM, family history diabetes mellitus; BMI, body mass index; GDM, gestational diabetes; oral glucose tolerance test, OGTT; NS, not significant.

**Table 2:** Comparison of women (*n* = 265) with normal OGTT, impaired OGTT (IGT/IFG) and those with significantly impaired OGTT (T2DM).

	Normal (Mean ± SD)	Abnormal (Mean ± SD)		*p* Value
		IGT/IFG (95% CI)	T2DM (95% CI)	
*n* (%)	210 (79.2)	55 (20.8)	10 (3.8)	
Age (years)	31.0 (30.2–31.8)	29.8 (28.3–31.3)	31.5 (27.7–35.3)	NS
FH DM	47.1%	78.0%	50%	NS
BMI	28.9 (28.1–29.7)	27.5 (25.7–29.3)	30.8 (26.4–35.1)	NS
Previous GDM	27.9%	31.6%	37.5%	<0.01 *
Gravida	3.4 (3.1–3.7)	2.7 (2.1–3.3)	3.60 (1.9–5.3)	NS
Abortions	2.2 (1.1–2.8)	1.46 (0.8–2.2)	1.25 (0.5–2.1)	NS
Birth Weight	3252.9 (3001.7–3504.1)	3186.5 (3018.3–3354.7)	3265.5 (2946.9–3584.1)	NS
Glucose tolerance week	23.9 (22.9–24.9)	23.8 (21.3–26.4)	24.2 (17.0–31.4)	NS
Gestational age at delivery (weeks)	38.3 (34.0–42.8)	38.4 (34.7–41.9)	37.4 (33.1–39.2)	NS
Fasting plasma glucose, mg/dL	90.3 (89.3–91.4)	100.4 (97.6–103.2)	127.1 (93.1–161.2)	<0.01
1 h post 75 g glucose, mg/dL	131.2 (120.6–141.8)	172.8 (128.4–139.4)	218.0 (116.4–319.7)	<0.01
2 h post 75 g glucose, mg/dL	105.6 (101.2–109.1)	131.7 (124.1–139.4)	215.9 (186.0–245.7)	<0.01

FH DM, family history diabetes mellitus; BMI, body mass index; GDM, gestational diabetes. IGT, impaired glucose tolerance; IFG, impaired fasting glucose; T2DM, type 2 diabetes mellitus. * Cramer’s V, 0.23; very small effect.

## Data Availability

The data presented in this study are available on request from the corresponding author. The data are not publicly available due to the confidential nature of the hospital data.

## References

[1] O’Sullivan J., Mahan C. (1964). Criteria for the oral glucose tolerance test in pregnancy. Diabetes.

[2] Casey B.M., Lucas M.J., Mcintire D.D., Leveno K.J. (1997). Pregnancy outcomes in women with gestational diabetes compared with the general obstetric population. Obstet. Gynecol..

[3] Wendland E.M., Torloni M.R., Falavigna M. (2012). Gestational diabetes and pregnancy outcomes-a systematic review of the World Health Organization (WHO) and the International Association of Diabetes inpregnancy study groups (IADPSG) diagnostic criteria. BMC Pregnancy Childbirth.

[4] Bellamy L., Casas J.P., Hingorani A.D., Williams D. (2009). Type 2 diabetes after gestational diabetes a systematic review and meta-analysis. Lancet.

[5] Vounzoulaki E., Khunti K., Abner S.C., Tan B.K., Davies M.J., Gillies C.L. (2020). Progression to type 2 diabetes in women with a known history of gestational diabetes: Systematic review and meta-analysis. BMJ.

[6] You H., Hu J., Liu Y., Luo B., Lei A. (2021). Risk of type 2 diabetes mellitus after gestational diabetes mellitus: A systematic review & meta-analysis. Indian J. Med. Res..

[7] Shen Y., Li W., Leng J., Zhang S., Liu H., Li W., Wang L., Tian H., Chen J., Qi L. (2019). High risk of metabolic syndrome after delivery in pregnancies complicated by gestational diabetes. Diabetes Res. Clin. Pract..

[8] Sun J., Kim G.R., Lee S.J., Kim H.C. (2021). Gestational diabetes mellitus and the role of intercurrent type 2 diabetes on long-term risk of cardiovascular events. Sci. Rep..

[9] American Diabetes Association Professional Practice Committee (2022). Classification and Diagnosis of Diabetes: Standards of Medical Care in Diabetes—2022. Diabetes Care.

[10] Ratner R.E., Christophi C.A., Metzger B.E. (2008). Diabetes Prevention Program Research Group. Prevention of diabetes in women with a history of gestational diabetes: Effects of metformin and lifestyle interventions. J. Clin. Endocrinol. Metab..

[11] Aroda V.R., Christophi C.A., Edelstein S.L., Zhang P., Herman W.H., Barrett-Connor E., Delahanty L.M., Montez M.G., Ackermann R.T., Zhuo X. (2015). The effect of lifestyle intervention and metformin on preventing or delaying diabetes among women with and without gestational diabetes: The Diabetes Prevention Program outcomes study 10-year follow-up. J. Clin. Endocrinol. Metab..

[12] Agarwal M.M., Dhatt G.S., Shah S.M. (2010). Gestational diabetes mellitus: Simplifying the international association of diabetes and pregnancy diagnostic algorithm using fasting plasma glucose. Diabetes Care.

[13] Agarwal M.M. (2020). Gestational Diabetes in the Arab Gulf Countries: Sitting on a Land-Mine. Int. J. Environ. Res. Public Health.

[14] Nielsen K.K., Kapur A., Damm P., de Courten M., Bygbjerg I.C. (2014). From screening to post-partum follow-up—The determinants and barriers for gestational diabetes mellitus (GDM) services, a systematic review. BMC Pregnancy Childbirth.

[15] Eggleston E.M., LeCates R.F., Zhang F., Wharam J.F., Ross-Degnan D., Oken E. (2016). Variation in Post-partum Glycemic Screening in Women with a History of Gestational Diabetes Mellitus. Obstet. Gynecol..

[16] Kjos S.L. (2007). After pregnancy complicated by diabetes: Postpartum care and education. Obstet. Gynecol. Clin. N. Am..

[17] Mcgovern A., Butler L., Jones S., Van Vlymen J., Sadek K., Munro N., Carr H., De Lusignan S. (2014). Diabetes screening after gestational diabetes in England. Br. J. Gen. Pract..

[18] Daly B., Toulis K.A., Thomas N., Gokhale K., Martin J., Webber J., Keerthy D., Jolly K., Saravanan P., Nirantharakumar K. (2018). Increased risk of ischemic heart disease, hypertension, and type 2 diabetes in women with previous gestational diabetes mellitus, a target group in general practice for preventive interventions: A population-based cohort study. PLoS Med..

[19] Ferrara A., Peng T., Kim C. (2009). Trends in postpartum diabetes screening and subsequent diabetes and impaired fasting glucose among women with histories of gestational diabetes mellitus: A report from the Translating Research into Action for Diabetes (TRIAD) Study. Diabetes Care.

[20] Nouhjah S., Shahbazian H., Amoori N., Jahanfar S., Shahbazian N., Jahanshahi A., Cheraghian B. (2017). Postpartum screening practices, progression to abnormal glucose tolerance and its related risk factors in Asian women with a known history of gestational diabetes: A systematic review and meta-analysis. Diabetes Metab. Syndr..

[21] Korpi-Hyövälti E., Laaksonen D.E., Schwab U., Heinonen S., Niskanen L. (2012). How can we increase postpartum glucose screening in women at high risk for gestational diabetes mellitus?. Int. J. Endocrinol..

[22] Hunt K.J., Conway D.L. (2008). Who returns for postpartum glucose screening following gestational diabetes mellitus?. Am. J. Obstet. Gynecol..

[23] Weinert L.S., Mastella L.S., Oppermann M.L., Silveiro S.P., Guimarães L.S., Reichelt A.J. (2014). Postpartum glucose tolerance status 6 to 12 weeks after gestational diabetes mellitus: A Brazilian cohort. Arq. Bras. de Endocrinol. Metabol..

[24] Benhalima K., Jegers K., Devlieger R., Verhaeghe J., Mathieu C. (2016). Glucose Intolerance after a Recent History of Gestational Diabetes Based on the 2013 WHO Criteria. PLoS ONE.

[25] Blatt A.J., Nakamoto J.M., Kaufman H.W. (2011). Gaps in diabetes screening during pregnancy and postpartum. Obstet. Gynecol..

[26] Ward R.J., Fryer A.A., Hanna F.W., Spencer N., Mahmood M., Wu P., Heald A.H., Duff C.J. (2021). Inadequate postpartum screening for type 2 diabetes in women with previous gestation diabetes mellitus: A retrospective audit of practice over 17 years. Int. J. Clin. Pract..

[27] Hjelm K., Bard K., Nyberg P., Apelqvist J. (2005). Swedish and Middle-Eastern-born women’s beliefs about gestational diabetes. Midwifery.

[28] Herath H., Herath R., Wickremasinghe R. (2017). Gestational diabetes mellitus and risk of type 2 diabetes 10 years after the index pregnancy in Sri Lankan women—A community based retrospective cohort study. PLoS ONE.

[29] Lowe W.L., Scholtens D.M., Lowe L.P., Kuang A., Nodzenski M., Talbot O., Catalano P.M., Linder B., Brickman W.J., Clayton P. (2018). Association of Gestational Diabetes With Maternal Disorders of Glucose Metabolism and Childhood Adiposity. JAMA.

[30] Lawrence J.M., Black M.H., Hsu J.W., Chen W., Sacks D.A. (2010). Prevalence and timing of postpartum glucose testing and sustained glucose dysregulation after gestational diabetes mellitus. Diabetes Care.

[31] Feig D.S., Chen E., Naylor C.D. (1998). Self-perceived health status of women three to five years after the diagnosis of gestational diabetes: A survey of cases and matched controls. Am. J. Obstet. Gynecol..

[32] Kilgour C., Bogossian F.E., Callaway L., Gallois C. (2019). Postnatal gestational diabetes mellitus follow-up: Perspectives of Australian hospital clinicians and general practitioners. Women Birth.

[33] Hjelm K., Berntorp K., Frid A., Aberg A., Apelqvist J. (2008). Beliefs about health and illness in women managed for gestational diabetes in two organisations. Midwifery.

[34] Pastore I., Chiefari E., Vero R., Brunetti A. (2018). Postpartum glucose intolerance: An updated overview. Endocrine.

